# Fine-Grained Personalized Data Aggregation Scheme with High Quality and Privacy Protection

**DOI:** 10.3390/s25216712

**Published:** 2025-11-03

**Authors:** Zhuoyue Xia, Raja Kumar Murugesan

**Affiliations:** School of Computer Science, Taylor’s University, Subang Jaya 47500, Malaysia; xiazhuoyue@sd.taylors.edu.my

**Keywords:** mobile crowd sensing, data aggregation, privacy protection, truth discovery, fine-grained, Paillier homomorphic encryption

## Abstract

Mobile crowd sensing (MCS) frequently relies on truth discovery to aggregate noisy, conflicting reports into reliable estimates. Existing approaches often either risk exposing user data or overlook heterogeneous privacy needs and task-specific reliability, limiting aggregation fidelity. This study presents a task-wise, personalized, privacy-preserving truth discovery framework that learns per-user, per-task weights to enable high-quality aggregation while protecting both location and data privacy. Structural privacy is realized via aggregate-only Paillier homomorphic encryption—only aggregate sums are decrypted at the cloud—and task-scoped unlinkable pseudonyms that prevent cross-task linkage. The design also supports fine-grained incentives, aligning rewards with task-level contributions without revealing raw readings or identities. Evaluations on real-world MCS temperature traces and simulated workloads show accuracy relative to a non-private baseline (MAE/RMSE on the order of $10^{-5}$), fast and stable convergence under a uniform stopping rule, and predictable scaling with users, tasks, and key sizes; cloud-side decryption is the dominant cost, whereas the iterative solver remains stable. Overall, personalized weighting combined with structural privacy delivers practical, high-quality aggregation for privacy-critical MCS deployments.

## 1. Introduction

The widespread adoption of mobile devices has made mobile crowd sensing (MCS) a practical paradigm for large-scale data collection. As a participatory sensing architecture, MCS can offer broader coverage, higher spatial–temporal resolution, and lower cost than traditional wireless sensor networks [1]. By assigning sensing tasks to registered participants, the system treats mobile users as sensing units and gathers the required measurements. MCS supports diverse applications, including health services [2], public safety [3], intelligent transportation [4], and environmental monitoring [5,6]. However, user-contributed measurements are often noisy or unreliable: participants may behave strategically to maximize rewards, and devices can suffer from calibration errors, sensor drift, or hardware faults. These subjective and objective factors necessitate truth discovery—the principled aggregation of multi-source reports to infer reliable estimates from conflicting or incomplete data.

In traditional data aggregation, to improve the quality of data aggregation, the common method is to design an effective incentive mechanism to attract and retain users to participate in data collection tasks and encourage them to submit high-quality data [7]. Typical approaches optimize participant behavior via game-theoretic design [8], auction-based payments [9], reputation systems [10], or psychological incentives [11]. For example, Bedogni and Montori put forward an opportunistic mobile crowd sensing system with privacy priority, which encourages workers to submit more accurate data through negotiation reward mechanism and k-anonymity evaluation, while considering privacy protection [12]. Edirimannage et al. proposes a quality-aware robust model aggregation framework for mobile crowdsourcing federated learning. Through model selection and adaptive incentive mechanism based on model performance, models can be efficiently aggregated in dependent and identically distributed data scenes and free occupants can be detected [13]. Cheng et al. introduced an innovative method to solve the problem of evaluating the quality of sensing data in the absence of reference information by integrating social psychology [11].

To evaluate data quality more accurately and realize high-quality data aggregation, truth discovery technology has been increasingly studied [14,15]. Liu et al. propose a privacy-preserving truth discovery scheme with enhanced reliability, aiming at solving the defects that the existing privacy-preserving truth discovery mechanism has abnormal values in the sensing data, which may significantly reduce the reliability of the algorithm and cannot guarantee strong privacy protection [16]. Cheng et al. propose a privacy protection and reputation-based truth discovery framework, which aims to generate the true value of sensing tasks with high accuracy, while protecting the privacy of sensing data, weights, and reputation values [17]. Zhang et al. propose an efficient and powerful privacy-preserving truth discovery scheme, which aims to protect users’ task privacy and data privacy at the same time [18]. Bai et al. propose an efficient and credible bilateral privacy protection truth discovery scheme, aiming at obtaining high-quality truth and preventing privacy leakage between workers and data requesters [19]. Through the above literature, we can find that when using truth discovery, we need to get in touch with the specific data of users, so the privacy protection of users also needs to be considered at the same time.

Traditional centralized architectures in MCS face well-known issues of reliability, data quality, and scalability/efficiency. To mitigate these limitations, recent studies leverage blockchain and distributed trust, combining novel consensus protocols with on-chain/off-chain storage and smart contracts. Huang et al. put forward a mobile crowd sensing platform, BlockSense, based on blockchain. Through the consensus protocol of “data proof” and homomorphic data disturbance, the system is decentralized, data is credible, privacy protection and incentive fairness are realized, and the verification efficiency is significantly improved [20]. Yu et al. put forward a privacy protection data aggregation and quality-driven incentive mechanism based on smart contract and IPFS, which ensures the fairness and credibility of data aggregation in mobile crowd sensing while realizing decentralization and reducing the storage cost on the chain [21]. Gu et al. propose a distributed trusted data transaction framework based on alliance blockchain [22]. Through Stackelberg game and smart contract, the fairness and efficiency of data transaction in mobile crowd sensing are realized while protecting privacy and reputation.

As mobile crowd sensing platforms scale and data volumes grow, attention has shifted to alleviating the load on centralized nodes. A prominent direction is to adopt edge/fog computing frameworks that push computation and aggregation closer to data sources [23]. Yang et al. propose a new “cloud enhancement-edge-terminal” MCS framework, which aims at assisting the edge server by using MCS idle users as edge nodes (ENs), thus enhancing the computing power, reducing the delay and energy consumption, and realizing efficient data aggregation [24]. Yan et al. propose a privacy-preserving multi-task data aggregation scheme for fog computing, which aims to solve the challenges brought by multi-task concurrency in urban environment, especially to protect the privacy of users’ decision to accept tasks and the privacy of user data and aggregation results [25]. Yan et al. propose a verifiable, reliable, and privacy-preserving data aggregation scheme, aiming at solving the calculation, communication, and storage challenges of the traditional model in fog-assisted mobile crowd sensing (FA-MCS), and coping with the risk of privacy disclosure of user data and aggregation results during data aggregation and the possibility that untrusted servers and fog nodes (FNs) may damage the correctness of aggregation results [26].

Designing a data aggregation scheme must balance truth discovery for quality, privacy protection, and computational efficiency. The proposed framework integrates Paillier homomorphic encryption with an edge–cloud architecture (fog nodes, FNs, cooperating with a cloud service provider, CSP) to enforce structural privacy while enabling scalable aggregation. To capture heterogeneous reliability, the method learns per-user, per-task contributions, enabling fine-grained, personalized aggregation and incentives. The main contributions are as follows:

(1) A fine-grained personalized aggregation scheme is presented that combines truth discovery with Paillier aggregate-only decryption; the FN–CSP distributed structure protects user data and locations while supporting efficient encrypted aggregation.
(2) The framework estimates each user’s contribution for each task, enabling fine-grained rewards aligned with task-level reliability and thereby promoting sustained high-quality participation.
(3) Experiments on real datasets demonstrate accurate aggregation under privacy constraints, with fast, stable convergence and predictable scalability across users, tasks, and key sizes.

## 2. Preliminaries

### 2.1. Truth Discovery

Truth discovery refers to algorithmic procedures that infer reliable information from incomplete or conflicting multi-source data. In MCS and related settings, heterogeneous user quality, noise, and conflicts are common; hence, both per-user reliability weights and object truths must be estimated jointly.

An alternating optimization procedure is adopted for truth discovery, wherein per-task user weights and task truths are updated in turn until a normalized convergence criterion is satisfied. Let T denote the task set with ∣T∣=T, and let $U_{t}\subseteq \left\{1,\ldots ,U\right\}\;$ denote the users who performed task t with $|U_{t}|=U_{t}^{\prime }$. For each claim $x_{t}^{u}$($t\in T,\;u\in U_{t}$), the distance dist(⋅,⋅) measures the discrepancy to the current truth $x_{t}^{\left(r\right)}$. At iteration r, user reliability weights are computed by a log-ratio model and normalized per task; truths are then updated as weighted averages of the corresponding claims. A small constant δ is included in denominators and logarithms for numerical stability, and per-task weight vectors are normalized each round to avoid scale drift. Convergence is declared when the normalized $l_{2}$ change in the truth vector falls below ε or when the maximum number of rounds $K_{max}$ is reached.

For a task t, user u provides a claim $x_{t}^{u}$. The truth $x_{t}^{*}$ is the latent factual value of object t, estimated by aggregating claims. The weight $w_{t}^{u}$ indicates user u’s reliability on task t; larger weights yield greater influence in aggregation.

Let the task set be T with ∣T∣=T, and the user pool {1,…,U}. For task t∈T, let $U_{t}\subseteq \{1,\ldots ,U\}$ denote participating users with $|U_{t}|=U_{t}^{\prime }$. Truth discovery alternates between weight update and truth update until convergence, as detailed below.

For computing the truth $x_{t}^{*}=(\sum_{u\in U_{t}}w_{t}^{u}x_{t}^{u})/(\sum_{u\in U_{t}}w_{t}^{u})$, the protocol supports an aggregate-only mode in which only the encrypted aggregates $S_{1,t}=\sum_{u\in U_{t}}w_{t}^{u}x_{t}^{u}$ and $S_{2,t}=\sum_{u\in U_{t}}w_{t}^{u}$ are decrypted to produce $x_{t}^{*}$. This is the default minimal-disclosure configuration. When deployments require individual-level trust management, incentive settlement, or auditable compliance, a controlled per-user weight option is available: weights are linked to pseudonymous identifiers with role separation, and threshold/range proofs can be used to limit information content. Both configurations yield identical $x_{t}^{*}$; the latter is enabled only when such functions are mandated.

**Weight update.** With the current truth estimate $x_{t}$ fixed, discrepancies and their sum are

(1) $$d_{t}^{u}=dist\left(x_{t}^{u},x_{t}\right),S_{t}=\sum_{u=1}^{U^{\prime }}\;d_{t}^{u}$$

A log-ratio weighting with ε-smoothing is adopted for numerical stability and robustness:

(2) $${\tilde{w}}_{t}^{u}=log\left(\frac{S_{t}+\delta }{d_{t}^{u}+\delta }\right),\delta >0$$

followed by nonnegativity clipping and per-task normalization.

(3) $$w_{t}^{u}\leftarrow max\left({\tilde{w}}_{t}^{u},0\right),w_{t}^{u}\leftarrow \frac{w_{t}^{u}}{max\left(\sum_{v=1}^{U^{\prime }}\;w_{t}^{v},\delta \right)}$$

This mapping is monotone, decreasing in $d_{t}^{u}$, mitigates instability at zero/near-zero discrepancies via δ, and reduces the influence of outliers or heavy-tailed errors through logarithmic compression and clipping.

**Truth Update.** With weights fixed, the truth of task t is updated by a weighted average:

(4) $$x_{t}\leftarrow \frac{\sum_{u=1}^{U^{\prime }}\;w_{t}^{u}x_{t}^{u}}{max\left(\sum_{u=1}^{U^{\prime }}\;w_{t}^{u},\delta \right)}$$

**Stopping criterion and output.** Iterations terminate when the normalized change in the truth vector falls below a tolerance or when a maximum number of rounds is reached:

(5) $$\Delta =\frac{{\left(\sum_{t=1}^{T}\;{\left|x_{t}^{\mathrm{new}\;}-x_{t}^{\mathrm{old}\;}\right|}^{2}\right)}^{1/2}}{max\left(1,{\left(\sum_{t=1}^{T}\;{\left|x_{t}^{\mathrm{old}\;}\right|}^{2}\right)}^{1/2}\right)}<\varepsilon \;\;\mathrm{or}\;\;r\geq K_{max}$$

At termination, the final ground truths are set to $x_{t}^{*}=x_{t}$ for all t. Users whose claims are closer to the converged truths receive higher learned weights and thus larger aggregation influence.

The safeguards in Equations (2) and (3) (ε-smoothing, clipping, per-task normalization) are part of the update rule and ensure stability in pathological cases. δ is set to a negligible value and clipping is rarely activated, while remaining available to guarantee well-defined behavior under zero/near-zero discrepancies or heavy-tailed errors.

Complexity and stability note. Per iteration, the weight update and truth update steps for task t each cost $O(|U_{t}|)$; hence, one full round costs $O(\sum_{t\in T}|U_{t}|)$. With the adopted safeguards—δ-smoothing, nonnegativity clipping, and per-task normalization in (2) and (3)—the alternating schedule implements a monotone descent on a weighted squared-error surrogate under the fixed stopping rule in (5). Because Paillier aggregation is additively exact within range, encrypted updates are numerically equivalent to plaintext updates provided no modulus wrap-around occurs. A sufficient range condition is $|\;\sum_{u\in U_{t}}w_{t}^{u}x_{t}^{u}|<n/2$ and $|\;\sum_{u\in U_{t}}w_{t}^{u}|<n/2$ for all t, which holds under bounded domains for $x_{t}^{u}$ and normalized $w_{t}^{u}$. The truth discovery algorithm is given in Algorithm 1.
**Algorithm 1:** Truth Discovery**Inputs.** Task set T with ∣T∣=T; user pool {1,…,U}; per-task user sets $U_{t}\subseteq \{1,\ldots ,U\}$ with $|U_{t}|=U_{t}^{\prime }$; claims $\left\{x_{t}^{u}:t\in T,\;u\in U_{t}\right\}$; distance dist(⋅,⋅); tolerance ε; maximum rounds $K_{max}$; stability constant δ>0.
**Outputs.** Ground truths $\left\{x_{t}^{*}:t\in T\right\}$ and weights $\left\{w_{t}^{u}:t\in T,\;u\in U_{t}\right\}$.
**Initialization.** For each t∈T, set the initial truth $x_{t}$ to the sample mean of $\left\{x_{t}^{u}:u\in U_{t}\right\}$; set $w_{t}^{u}=1/U_{t}^{\prime }$ for all $u\in U_{t}$.
**Repeat** for r=0,1,… until the stopping rule in (5) is satisfied or $r\geq K_{max}$:     **Weight update (given truths).**    For each t∈T, compute discrepancies and their sum as in (1); obtain raw log-ratio weights with ε-smoothing as in (2); then apply nonnegativity clipping and per-task normalization as in (3). If the normalization sum is zero, set $w_{t}^{u}=1/U_{t}^{\prime }$ for all $u\in U_{t}$.    **Truth update (given weights).**    For each t∈T, update the truth by the weighted average in (4).    **Stopping check.**    Evaluate the normalized change Δ of the truth vector according to (5); if Δ<ε, terminate.
**Return.** Set $x_{t}^{*}=x_{t}$ for all t∈T and output the corresponding weights $\left\{w_{t}^{u}\right\}$.

### 2.2. Paillier Homomorphic Encryption

Paillier homomorphic encryption is an additive homomorphic public-key scheme that supports linear operations on ciphertexts, enabling privacy-preserving aggregation without decryption. In this work, the standard g=1+n instantiation (as in [24]) is adopted, which yields the encryption map in (6). Compared with the generic form $g^{m}r^{n}\;\;mod\;\;n^{2}$ used in some truth discovery systems [27], the g=1+n choice eliminates one modular exponentiation at encryption time: the factor $g^{m}\;mod\;n^{2}$ reduces to the linear term (1+mn), computable via a single modular multiplication, while the randomization $r^{n}$ (here realized as $h^{nr_{k}}$) still requires one exponentiation. As modular exponentiation dominates client-side cost at typical key sizes, this reduction improves computational cost and scalability for mobile devices while leaving FN-side aggregation and CSP-side decryption unchanged. Security guarantees are unchanged (semantic security under the decisional composite residuosity—DCR—assumption); thus, the chosen instantiation is more effective in this context because it achieves the same security and functionality with fewer modular exponentiations.

#### 2.2.1. The Basic Principle of Paillier Encryption

Let $m\in Z_{n}$ be a plaintext. The public key is $pk=(n,h^{\;n})$, where n=pq for large primes p,q, and $h\in Z_{n^{2}}^{*}$ serves as a randomness base to ensure semantic security. To encrypt, sample a per-ciphertext randomizer $r_{k}$ and output

(6) $$c=E_{pk}(m)=(1+n\cdot m)\cdot h^{n\cdot r_{k}}\;mod\;n^{2}$$

Here, h∈$Z_{n^{2}}^{*}$ is a public key parameter that serves as a random base to prevent the determinism of ciphertext. This expression is algebraically equivalent to the canonical Paillier form $g^{m}r^{n}\;mod\;n^{2}$; here (1+n⋅m) plays the role of $g^{m}$, and $h^{\;n\cdot r_{k}}$ corresponds to the randomization term $r^{n}$.

The private key is sk=λ=(p−1)(q−1). Define L(u)=(u−1)/n. The decryption of $c\in Z_{n^{2}}^{*}$ recovers

(7) $$m=D_{sk}\left(c\right)=\frac{\left(c^{\lambda }mod\;n^{2}-1\right)}{n}\cdot \lambda^{-1}mod\;n$$

i.e., $m=L(c^{\lambda }\;mod\;n^{2})\cdot \lambda^{-1}\;mod\;n$. The randomization $h^{\;n\cdot r_{k}}$ yields semantic security under the DCR assumption.

#### 2.2.2. Homomorphism Property

Paillier supports additive homomorphism and scalar multiplication over plaintexts:

(8) $$E_{pk}\left(m_{1}\right)\cdot E_{pk}\left(m_{2}\right)=E_{pk}\left(m_{1}+m_{2}\right),\;\;E_{pk}(m{)}^{a}=E_{pk}(am)(a\in Z)$$

These properties allow encrypted summation and weighted aggregation without exposing individual inputs.

## 3. Methods

### 3.1. Algorithm Framework

**Figure 1:**
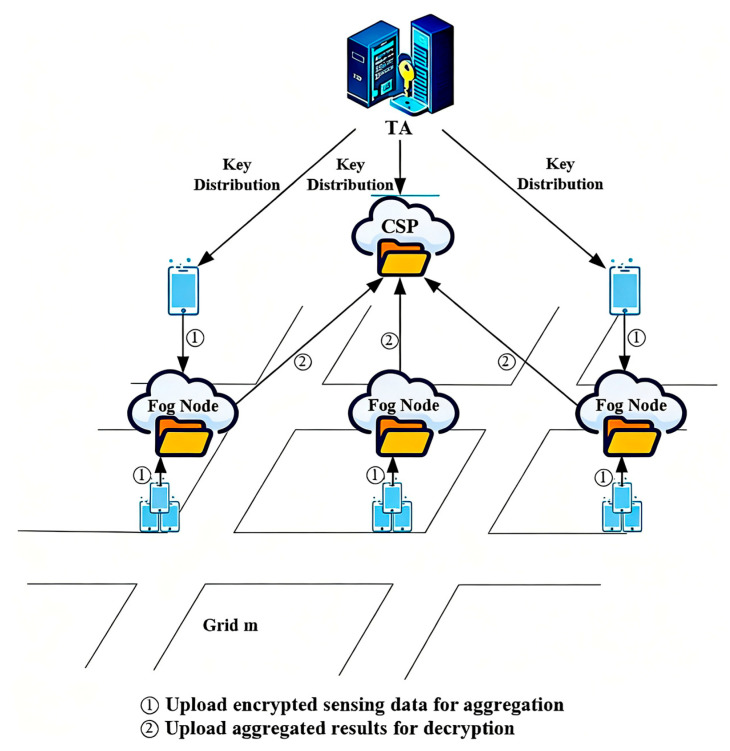
Algorithm frame diagram.

Considering privacy-preserving high-quality data aggregation, this study proposes a fine-grained personalized privacy-preserving high-quality data aggregation scheme, shown in Figure 1. The architecture comprises a trusted authority (TA), a CSP, FNs, a task requester (publisher), and task performers (users). Two message phases are indicated in the figure: ① upload encrypted sensing data to the FN; ② upload encrypted aggregates to the CSP for decryption.

TA: The TA is trusted only for setup. It generates the Paillier keypair, distributes the public key to clients, and provisions the secret key to the CSP inside protected hardware; its copy is erased thereafter. The TA does not handle user locations or runtime data and holds no identity–pseudonym linkage during operation.

CSP: The CSP orchestrates task publication and result delivery. Under the minimal-disclosure policy, the CSP decrypts only aggregates—$S_{1,t}=\sum_{u\in U_{t}}w_{t}^{u}x_{t}^{u}$ and $S_{2,t}=\sum_{u\in U_{t}}w_{t}^{u}$—to produce $x_{t}^{*}=S_{1,t}/S_{2,t}$. Raw readings and identities are never exposed to the CSP.

FNs: FNs relay and locally combine user-side ciphertexts, manage task-scoped pseudonyms for unlinkability, form adaptive user groups, and forward encrypted aggregates to the CSP. FNs have no decryption key; identity–pseudonym mappings are stored locally and scoped to the settlement window.

Task requester (publisher): Responsible for submitting task requests to CSP and collecting data collected by the task.

Task performer (users): Users receive tasks, encrypt their sensing data locally with the TA-issued public key, attach a fresh pseudonym ${pse}_{u,t}$ per task, and upload the ciphertexts to the assigned FN. Distances and weights used by the truth discovery loop are computed client-side and submitted in encrypted form.

### 3.2. Threat Model

The system comprises a TA that is trusted only for setup (key generation, parameter issuance, role separation), a CSP that is semi-honest (honest-but-curious) and has no access to identity mappings, FNs that are semi-honest and handle ciphertext forwarding and pseudonymous identifiers without decryption keys, and users who may be curious or adversarial (submitting noisy or strategically manipulated claims $x_{t}^{u}$). The baseline assumes no collusion between CSP and FNs; stronger collusions considered include CSP↔FN, CSP↔TA, FN↔Users, and TA↔CSP↔FN. Privacy and security properties are defined as follows: C1 confidentiality of raw claims $x_{t}^{u}$ from CSP and FNs; C2 unlinkability between per-user weights $w_{t}^{u}$ and real identities through pseudonymous identifiers and role separation; C3 correctness of outputs; and C4 auditability/policy-compliant settlement using threshold/range proofs that avoid revealing exact $w_{t}^{u}$ when unnecessary. The cryptographic design defaults to aggregate-only decryption, revealing only $S_{1,t}=\sum_{u\in U_{t}}w_{t}^{u}x_{t}^{u}$ and $S_{2,t}=\sum_{u\in U_{t}}w_{t}^{u}$ to compute $x_{t}^{*}$; an optional controlled per-user mode may disclose selected $w_{t}^{u}$ (pseudonymous, policy-scoped, with range/threshold proofs) for trust management and incentive settlement, and both configurations yield identical $x_{t}^{*}$.

Table 1 summarizes which properties hold under different collusion patterns; the baseline (no collusion) preserves all C1–C4. Under CSP↔FN, C2 may degrade due to combined views; mitigations include threshold decryption across independent authorities and hardware isolation for identity mapping. Under FN↔Users, C1 may fail for colluding users while other users remain protected; pseudonym rotation and visibility rate-limiting reduce leakage. Under CSP↔TA, role separation preserves C1–C4. Under full infrastructure collusion (TA↔CSP↔FN), C1–C2 cannot be guaranteed, whereas C3–C4 can be retained via proof-carrying summaries and attestations. Transport-layer threats (replay, tamper, identity forgery) are orthogonal and can be addressed by standard MAC/signcryption without affecting the above properties.

System setup assigns Paillier keys as follows: the TA generates the keypair, publishes the public key to clients, and provisions the secret key to the CSP within protected hardware; the TA erases its copy thereafter. Decryption is CSP-only and follows the minimal-disclosure policy.

Location privacy is provided by a structural, non-perturbative design that combines task-level unlinkable pseudonyms with role separation so that the CSP never receives location fields. For each user u and task t, a fresh pseudonym ${pse}_{u,t}$ is generated; identities and location metadata are maintained only at the FN side for routing, whereas the CSP processes encrypted submissions and bounded weights without any location attributes. The guarantee is not differential privacy—no noise is added and thus no ε budget is claimed; the security parameter is that of the underlying cryptosystem, denoted λ.

Cross-task deanonymization is mitigated by task-scoped, unlinkable pseudonyms generated at the FN. For each user u and task t, the FN samples a nonce $\eta_{u,t}\overset{\$}{\leftarrow }\{0,1{\}}^{\lambda }$ and derives a fresh pseudonym

(9) $${pse}_{u,t}={PRF}_{K_{u}}\left(t\left\|\eta_{u,t}\right\|{\;\mathrm{salt}\;}_{\mathrm{epoch}\;}\right)$$

and stores the identity–pseudonym mapping locally for the settlement window only. The CSP receives $\left({pse}_{u,t},\;\mathrm{ciphertext},\;w_{t}^{u}\right)$ without identity or location fields. The per-task nonce and epoch salt ensure non-determinism; role separation (no identity mapping at the CSP) preserves unlinkability. Operational safeguards—minimum-crowd batching ($k_{min}$) and time-windowed reporting with randomized delay in [0,ΔT]—reduce side-channel linkage.

Under the non-collusion baseline (CSP↔ /FN), task-level unlinkability holds: for any user u and any two tasks $t_{1}\neq t_{2}$, any PPT adversary A observing the CSP’s view satisfies

(10) $$\left.\left|Pr\left[A\left({\;\mathrm{View}\;}_{CSP};{\;\mathrm{pse}\;}_{u,t_{1}},{\;\mathrm{pse}\;}_{u,t_{2}}\right)=1\right]-\frac{1}{2}\right.\right|\leq negl(\lambda )$$

i.e., deciding whether ${pse}_{u,t_{1}}$ and ${pse}_{u,t_{2}}$ belong to the same user is no better than random guessing. Moreover, location non-disclosure to the CSP holds: for any two user-location datasets $L,L^{\prime }$ of the same shape

(11) $${View}_{CSP}(L)\approx_{c}{View}_{CSP}\left(L^{\prime }\right)$$

So the CSP’s transcript distribution is computationally indistinguishable on L vs. $L^{\prime }$ because location fields are never transmitted to, nor stored by, the CSP. Under stronger collusion (CSP↔FN), unlinkability can degrade via combined views; mitigations include role separation with independent authorities, threshold decryption, and range/threshold proofs bound to pseudonyms.

The scheme provides structural (computational) privacy rather than differential privacy; no ε-budget or privacy–utility curves are claimed. Guarantees are stated via indistinguishability games. Under the non-collusion baseline (CSP↔ /FN), for any user u and tasks $t_{1}\neq t_{2}$, a PPT adversary observing the CSP’s view cannot decide whether ${\mathrm{pse}}_{\left(u,t_{1}\right)}$ and ${\mathrm{pse}}_{\left(u,t_{2}\right)}$ belong to the same user with advantage better than random guessing: $|Pr[A({\mathrm{View}}_{\mathrm{CSP}};{\mathrm{pse}}_{\left(u,t_{1}\right)},{\mathrm{pse}}_{\left(u,t_{2}\right)})=1]-\frac{1}{2}|\leq \mathrm{negl}(\lambda ).$ For any two location datasets $L,L^{\prime }$ of the same shape, the CSP’s transcript distributions are computationally indistinguishable, ${\mathrm{View}}_{\mathrm{CSP}}(L)\approx_{c}{\mathrm{View}}_{\mathrm{CSP}}(L^{\prime })$, since location fields are never transmitted to or stored by the CSP. Quantitative privacy-loss metrics and empirical leakage audits are out of scope for this work and are deferred to future research.

The core protocol assumes semi-honest users who correctly compute and encrypt their per-task distances; explicit defenses against input manipulation are out of scope of the main design. The scheme remains compatible with optional zero-knowledge safeguards (range proofs and distance–ciphertext consistency proofs) that preserve privacy and can be verified on ciphertexts; deployment details and overhead considerations are deferred to future work. The TA is trusted only for setup (key issuance under protected hardware and subsequent erasure). The CSP adheres to minimal-disclosure and decrypts aggregates ∑w and ∑wx only, never raw readings. To reduce single-point trust, the design is compatible with t-of-m threshold Paillier and multi-authority pseudonym issuance.

Message authenticity and freshness are enforced by standard transport and application controls: links between users, FNs, and the CSP run over TLS 1.3 with AEAD, and application messages are signcrypted (or MACed) under TA-provisioned keys. Each message embeds $\left(\mathrm{task}\_\mathrm{id},{pse}_{u,t},\mathrm{seq},\mathrm{nonce},\mathrm{timestamp}\right)$; receivers validate signatures/MACs, enforce a freshness window ΔT with a replay cache per pseudonym, and reject duplicate (seq,nonce) tuples. Tamper-evident logs (hash-chained, time-stamped) are maintained at FNs/CSP for audit. Keys follow periodic rotation and revocation via the TA. These controls are standard for deployment and are orthogonal to the cryptographic aggregation protocol analyzed in this work.

### 3.3. Design Details

#### 3.3.1. Initial Stage

The system comprises a TA, a CSP, FNs, and users. The TA is trusted only for setup. During initialization, the TA generates and distributes cryptographic keys, while task-scoped pseudonyms are provided at the FN side when a user enrolls in a task (to preserve cross-task unlinkability).

Given a security parameter K(key length), select two primes p,q with ∣p∣=∣q∣=K. Compute n=pq,λ=(p−1)(q−1), choose $h\in Z_{n^{2}}^{*}$. And form the public key to the CSP within protected hardware, which erases its own copy thereafter.

For each user k and task t, the responsible FN assigns a fresh task-level pseudonym ${pse}_{k,t}$ and keeps the identity–pseudonym mapping locally for the settlement window only; the CSP receives ciphertexts and bounded weights associated with ${pse}_{k,t}$ but no identity or location fields.

#### 3.3.2. Iterative Stage

Let the task set be $Task=\{t_{1},t_{2},\ldots ,t_{i}\}$. Each task $t_{i}$ is area-related and managed by an FN. After generating an initial truth $x_{\left(t_{i}\right)}^{*}$ for each task, the CSP disseminates $x_{\left(t_{i}\right)}^{*}$ via the corresponding FNs to their users.

Step 1: Fog node FN sends the task $t_{i}$ to the user $u_{k}$, k represents the user number and is an integer. The user $u_{k}$ calculates the distance $d_{t_{i}}^{u_{k}}=dist(x_{t_{i}}^{u_{k}},x_{t_{i}}^{*})$ between the user’s sensing data and the initial true value after collecting the sensing data $x_{t_{i}}^{u_{k}}$. It should be specially stated here that the data collected by MCS is divided into continuous data and discrete data, and the calculation for different data distances is different. For continuous data, use the following formula:

(12) $$dist(x_{t_{i}}^{u_{k}},x_{t_{i}}^{*})={\left(x_{t_{i}}^{u_{k}}-x_{t_{i}}^{*}\right)}^{2}$$

For discrete data, it can be expressed as vector $x_{t_{i}}^{u_{k}}$ = (0, …, 1, 0, …, 0), where the selected parameter is 1 and the rest are 0. The distance calculation formula is

(13) $$dist(x_{t_{i}}^{u_{k}},x_{t_{i}}^{*})={\left(x_{t_{i}}^{u_{k}}-x_{t_{i}}^{*}\right)}^{T}(x_{t_{i}}^{u_{k}}-x_{t_{i}}^{*})$$

The user $u_{k}$ selects a random value $r_{k}$ and saves it separately, and then encrypts $d_{t_{i}}^{u_{k}}$ with the following formula:

(14) $$E_{pk}(d_{t_{i}}^{u_{k}})=(1+n\cdot d_{t_{i}}^{u_{k}})\cdot h^{n\cdot r_{k}}mod\;n^{2}$$

The pair $\left({pse}_{k,t_{i}},\;E_{pk}(d_{\left(t_{i}\right)}^{\left(u_{k}\right)})\right)$ is uploaded to the FN.

Step 2: After receiving the distance encrypted data of $U^{\prime }$ users for task $t_{i}$, the fog node FN aggregates these data according to the homomorphism of Paillier encryption:

(15) $$E_{d}=\;\prod_{k=1}^{U^{\prime }}E_{pk}(d_{t_{i}}^{u_{k}})mod\;n^{2}\;=\;(1+n\cdot \sum_{k=1}^{U^{\prime }}d_{t_{i}}^{u_{k}})\cdot h^{n\cdot \sum_{k=1}^{U^{\prime }}r_{k}}mod\;n^{2}$$

The FN forwards the aggregate $E_{d}$ to the CSP.

Step 3: After CSP obtains the aggregated encrypted data $E_{t_{i}}$, it decrypts the aggregated data by using the private key sk, that is, λ = (p − 1) (q − 1):

(16) $${Sum}_{d}=D_{sk}\left(E_{d}\right)=\frac{E_{d}^{\lambda }mod\;n^{2}-1}{n}\cdot \lambda^{-1}mod\;n=\sum_{k=1}^{U^{\prime }}\;d_{\left(t_{i}\right)}^{\left(u_{k}\right)}$$

The value ${Sum}_{d}$ is returned to users via the FN.

Step 4: Assuming semi-honest clients, each user $u_{j}$ updates the weight for task $t_{i}$ locally using ${Sum}_{d}$ and the user-specific distance $d_{\left(t_{i}\right)}^{\left(u_{j}\right)}$, according to Equations (2) and (3) (log-ratio reweighting with δ-smoothing, clipping, and per-task normalization). The user then prepares two encrypted terms for aggregation

(17) $$E_{pk}\left(w_{\left(t_{i}\right)}^{\left(u_{j}\right)}\cdot x_{\left(t_{i}\right)}^{\left(u_{j}\right)}\right),E_{pk}\left(w_{\left(t_{i}\right)}^{\left(u_{j}\right)}\right)$$

and uploads them (together with ${pse}_{u_{j},t_{i}}$) to the FN. After all $U^{\prime }$ users submit, the FN computes the two aggregates

(18) $$\prod_{j=1}^{U^{\prime }}\;E_{pk}\left(w_{\left(t_{i}\right)}^{\left(u_{j}\right)}\cdot x_{\left(t_{i}\right)}^{\left(u_{j}\right)}\right),\prod_{j=1}^{U^{\prime }}\;E_{pk}\left(w_{\left(t_{i}\right)}^{\left(u_{j}\right)}\right),$$

and forwards the results to the CSP.

Step 5: The CSP only decrypts the two aggregates

(19) $${Sum}_{wx}=D_{sk}\left(\prod_{j=1}^{U^{\prime }}\;E_{pk}\left(w_{\left(t_{i}\right)}^{\left(u_{j}\right)}x_{\left(t_{i}\right)}^{\left(u_{j}\right)}\right)\right)$$

(20) $${Sum}_{w}=D_{sk}\left(\prod_{j=1}^{U^{\prime }}\;E_{pk}\left(w_{\left(t_{i}\right)}^{\left(u_{j}\right)}\right)\right)$$

and updates the truth by the weighted average:

(21) $$x_{\left(t_{i}\right)}^{*}\leftarrow \frac{{Sum}_{wx}}{max\left({Sum}_{w},\delta \right)}$$

**Figure 2:**
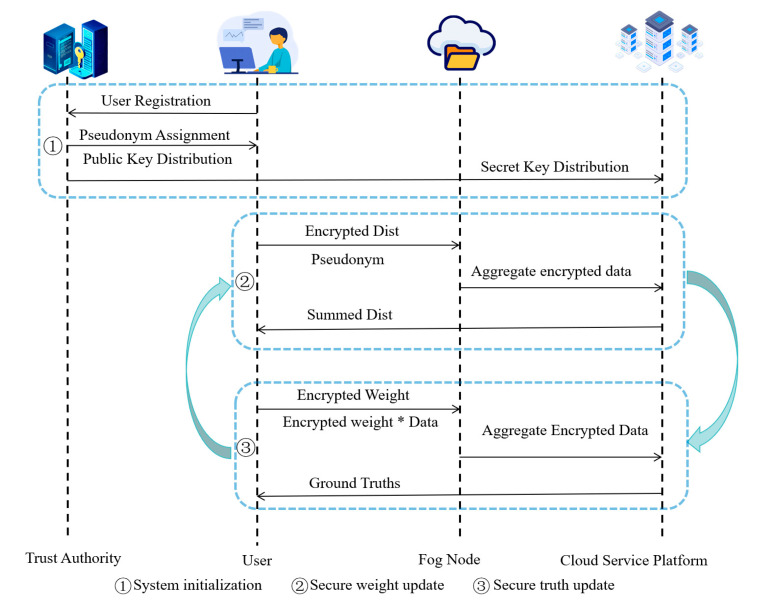
Algorithm flow chart (Note: All arrows assume TLS 1.3 + AEAD; application messages carry (seq, nonce, timestamp) and are signcrypted with TA-provisioned keys; receivers enforce replay checks within a window ΔT).

After each truth update at the CSP, the updated truth values $x_{\left(t_{i}\right)}^{*}$ are disseminated to the corresponding users via their FNs. The protocol then repeats phases ② secure weight update and ③ secure truth update (as labeled in Figure 2) until the normalized convergence criterion in Equation (5) is satisfied.

As shown in the figure, Phase ① performs system initialization (user registration, task-scoped pseudonym assignment, public/secret-key distribution). Phase ② executes the secure weight update loop: users submit encrypted distances and pseudonyms to the FN, which aggregates and forwards encrypted sums to the CSP; the CSP decrypts only the aggregates and returns the summed distance to users for local reweighting. Phase ③ executes the secure truth update loop: users submit $E_{pk}(w_{t}^{u}x_{t}^{u})$ and $E_{pk}(w_{t}^{u})$; the FN aggregates, the CSP decrypts the two totals, and $x_{t}^{*}$ is updated by Equation (4). The two phases iterate until convergence.

At convergence, each user u obtains per-task reliability weights $\{w_{t}^{u}{\}}_{t\in T_{u}}$ for the tasks actually performed; for $t\notin T_{u}$, the weight is set to 0. For convenience, define the (pseudonymous) weight vector

(22) $$W_{u}=\left(w_{t_{1}}^{u},w_{t_{2}}^{u},\ldots ,w_{t_{M}}^{u}\right),w_{t}^{u}=0\;\;\;\mathrm{if}\;t\notin T_{u}$$

where $t_{1},\ldots ,t_{M}$ enumerate the task universe. This summary is associated with the user’s task-level pseudonyms $\left\{{pse}_{u,t}\right\}$ as maintained at the FN.

Then, the user gives the pseudonym ${pse}_{k}$ and the weight set $W_{t_{i}}^{u_{k}}$ to TA, and TA knows the reward of each task, and rewards the user according to different weights, thus realizing fine-grained reward, prompting subsequent users to submit fine-grained data, promoting data aggregation results and obtaining high-quality data.

## 4. Experimentation

The existing work [28] mainly considers all factors, regardless of the task type. Different from the existing work, this scheme is a fine-grained personalized high-quality privacy protection data aggregation scheme, considering the specific contribution of each user to each task. The scheme refines the contribution of each user and makes the reward more accurate. To verify the effectiveness, all experiments are conducted on the CRAWDAD dataset queensu/crowd_temperature, using 135 users with sensing data partitioned into 20 task dimensions. The system follows a client-side homomorphic encryption→fog node ciphertext multiplication→cloud-side (CSP) decryption-and-aggregation pipeline. Experiments run on Windows 11 Pro, Java JDK 17 (compiler: IntelliJ IDEA 2023.1.1), with an AMD Ryzen 75800H with Radeon Graphics CPU and 32 GB RAM. The iterative procedures use a maximum of $K_{max}$ = 50 iterations with a convergence tolerance of ε= 10^−10^.

### 4.1. User Weight

As shown in Table 2, the task-wise private variant achieves MAE = 1.33 × 10^−5^ and RMSE = 1.39 × 10^−5^ against the non-private truth baseline, whereas the global/unified private variant shows a substantially larger error (MAE ≈ 4.17 × 10^−2^, RMSE ≈ 5.29 × 10^−2^) and requires many more iterations (37 vs. ≤12). This gap indicates that modeling user contributions per task/dimension avoids the bias induced by a single global weight and preserves the fidelity of truth discovery when operating under homomorphic encryption.

**Figure 3:**
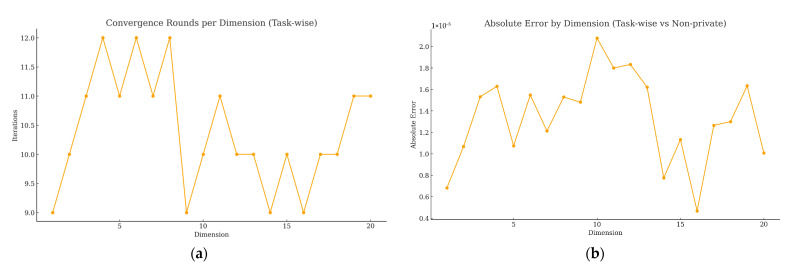
Per-dimension convergence and accuracy of task-wise private weighting. (**a**) Convergence rounds per dimension (task-wise private); (**b**) absolute error by dimension (task-wise vs. non-private).

Figure 3a reports the convergence rounds per dimension for the task-wise scheme. All 20 dimensions converge between 9 and 12 iterations (mean 10.4, ε = 10^−10^), showing fast and stable convergence under a uniform stopping rule. Figure 3b plots the absolute error per dimension (vs. the non-private baseline). Errors remain within the 10^−5^ band across all dimensions, with small local peaks around Dims 10–12, but no sustained drifts or periodic patterns. A dimension-wise correlation analysis confirms that the converged truths from task-wise and non-private methods are nearly perfectly aligned (Pearson r≈1.0), i.e., homomorphic aggregation introduces negligible numerical distortion. We also observe a moderate positive correlation between absolute error and iteration counts (r≈0.46): dimensions that require one or two extra rounds tend to exhibit slightly larger (still tiny) absolute deviations, which is consistent with mild heterogeneity in those tasks rather than algorithmic instability.

**Figure 4:**
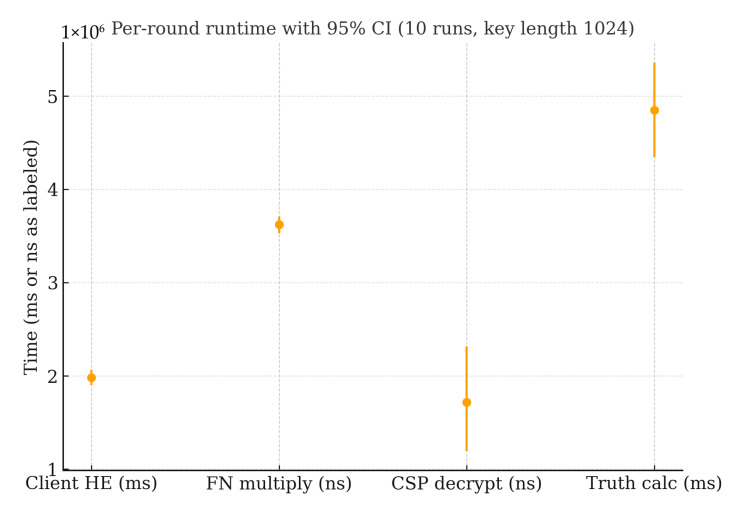
Per-round runtime with 95% confidence intervals (10 runs, key length 1024).

To complement the point estimates in Table 2, and Figure 3 and Figure 4 report 95% confidence intervals obtained by bootstrap over task dimensions (10,000 resamples) using the per-dimension residuals under the non-private baseline and the task-wise private variant. The intervals are narrow for both MAE and RMSE-derived stage times, indicating low dispersion across the 20 task dimensions under the uniform stopping rule $\left(\varepsilon =10^{-10},K_{max}=50\right)$. The confidence bands corroborate that the task-wise private variant tracks the non-private oracle baseline with deviations at the $10^{-5}$ level, consistent with the per-dimension absolute error band shown in Figure 3. No systematic drift is observed across dimensions.

From a methodological standpoint, these results clarify why a global weight deteriorates performance: users’ contributions are heterogeneous across tasks; forcing a single weight systematically over-weights some users in certain tasks and under-weights them in others, amplifying estimation bias and slowing convergence as the algorithm spends more iterations compensating for mis-specified weights. In contrast, task-wise weighting captures per-task marginal contributions, yielding near-lossless accuracy relative to the non-private oracle and rapid convergence in all dimensions.

### 4.2. User Reward

**Figure 5:**
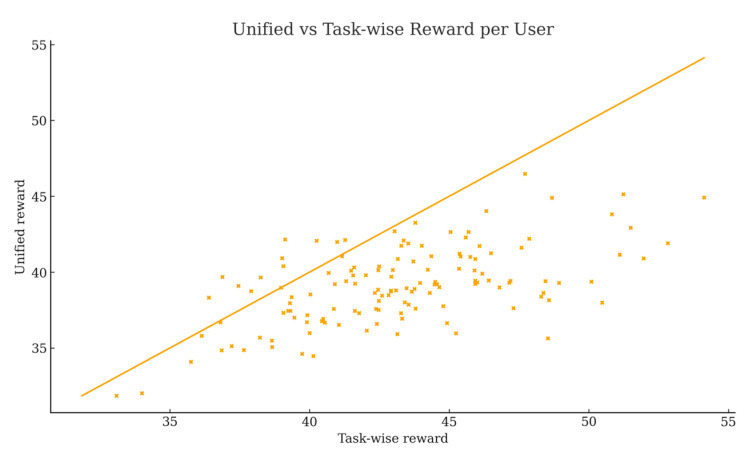
Unified vs. task-wise reward per user.

This section compares the per-user rewards obtained under two incentive designs: a task-wise scheme that aggregates rewards across 20 dimensions after per-task weighting, and a unified scheme that computes rewards using a single global weight. Figure 5 plots unified versus task-wise reward for each user together with the identity line; most points lie below the diagonal, showing that the unified scheme typically yields lower rewards than the task-wise computation. The mean task-wise reward is 43.18, while the unified mean is 39.11. Defining $\Delta =R^{uni}-R^{task}$, the average gap is −4.07 with a median of −4.06 and a standard deviation of 3.24. Only 8.89% of users receive higher rewards under the unified scheme, whereas 91.11% are under-rewarded; moreover, 35.6% exhibit ∣Δ∣>5, indicating practically meaningful deviations for a large subset of users.

**Figure 6:**
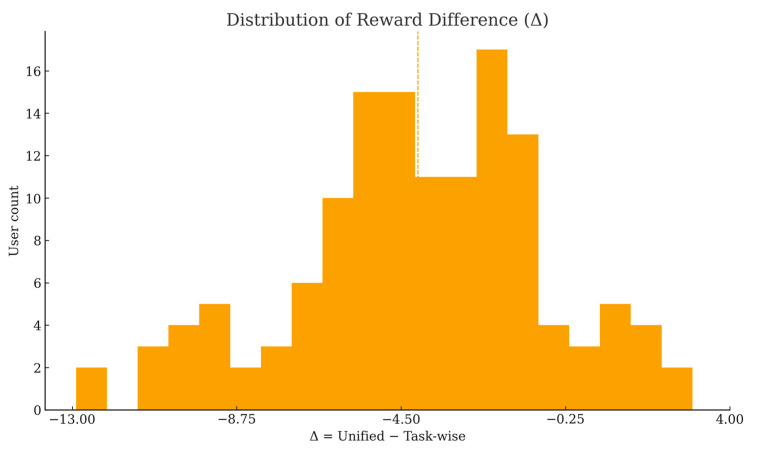
Distribution of reward difference (Δ).

The histogram in Figure 6 shows that Δ is left-skewed with mass concentrated between approximately −8 and −1. The dashed and dotted reference lines mark the sample mean and median, both close to −4, confirming a systematic negative bias of the unified scheme rather than random fluctuations. This pattern reflects cross-task heterogeneity in user contributions: when a user’s strength is concentrated in certain tasks, a single global weight fails to capture that value, leading to underpayment.

**Figure 7:**
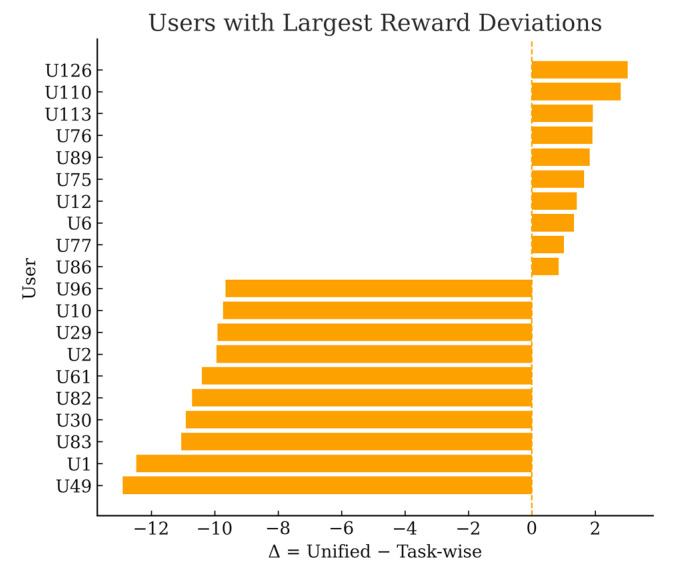
Users with largest reward deviations.

Figure 7 highlights the users with the largest deviations (top-10 underpaid and top-10 overpaid by the unified scheme). The horizontal layout with ASCII-only labels avoids any rendering ambiguity. The extremes reveal both sides of the problem: some users are substantially under-rewarded by the unified design (double-digit losses), while a small minority is over-rewarded, which risks reinforcing suboptimal sensing behavior. In contrast, the task-wise scheme aligns payments with per-task marginal contributions, improving fairness and incentive compatibility and thereby supporting higher-quality data aggregation over time.

### 4.3. Aggregation Efficiency

To assess the effect of the number of task dimensions on efficiency and convergence, this experiment varies the number of task dimensions while keeping the user set fixed. The results in Table 3 show a monotonic increase in total runtime as the dimensionality grows. The stage breakdown clarifies the bottleneck: CSP decryption dominates the end-to-end time among the three stages (client-side homomorphic encryption, fog node ciphertext multiplication, and cloud-side decryption), and its cost increases with the number of dimensions. The client-side homomorphic encryption cost is the second largest component, while the fog node ciphertext multiplication time remains comparatively small across all settings.

Convergence is stable across all dimension settings, requiring 9–10 rounds under the same stopping tolerance. The converged truth values remain numerically consistent (≈6.72389) across different dimensionalities, indicating that increasing the number of tasks does not compromise the numerical fidelity of truth discovery under the proposed pipeline. In combination with the stage breakdown, these observations reinforce that CSP decryption is the principal performance bottleneck, whereas edge computation and weight updates impose a relatively low overhead.

To assess the effect of the number of users on efficiency and convergence, this experiment varies the user cardinality while keeping the task dimension fixed at 20 and measures end-to-end runtime (minutes), per-stage costs (milliseconds), convergence rounds, and converged truths (Table 4). Total runtime increases predictably with user count—6.98 min (25 users)→9.64 min (45)→17.71 min (90)→28.11 min (135)—consistent with the growing cryptographic and aggregation workload. Micro-stage profiling further shows CSP decryption as the dominant per-stage cost across settings (≈13.54/11.92/12.12/14.50 ms for 25/45/90/135 users), followed by client-side homomorphic encryption (≈15.98/15.28/15.13/16.80 ms), while fog node ciphertext multiplication remains comparatively small (≈0.71/1.16/2.33/3.72 ms). Despite the higher load, convergence remains stable—14, 9, 10, and 9 rounds for 25/45/90/135 users, respectively—and the converged truths are numerically consistent (≈6.69, 7.07, 6.77, 6.72), indicating that scaling users does not degrade the numerical fidelity of truth discovery. Overall, the pipeline exhibits predictable scaling with user cardinality; CSP decryption emerges as the principal computational hotspot in per-stage terms, whereas edge computation and the iterative solver maintain robustness and fast convergence.

This experiment varies the homomorphic encryption key length while keeping the sensing workload fixed, and reports end-to-end runtime (minutes), per-stage costs (milliseconds), convergence rounds, and converged truth values (Table 5). Total runtime increases steeply with the key size—3.94 min at k = 512, 28.11 min at k = 1024, and 179.59 min at k = 2048—which is consistent with the super-linear growth of cryptographic cost (larger modulus/precision entails heavier big-integer arithmetic). Micro-profiling shows that CSP decryption dominates the per-stage cost across all key sizes (≈1.73/14.50/95.27 ms for k = 512/1024/2048), followed by client-side homomorphic encryption (≈2.37/16.80/108.56 ms), while fog node ciphertext multiplication remains the smallest contributor (≈1.40/3.72/10.65 ms). Despite the dramatic increase in runtime, convergence remains stable (≈9–10 rounds) and the converged truth stays numerically consistent (≈6.72389), indicating that larger key sizes mainly affect computational overhead rather than the numerical fidelity of truth discovery. Taken together, the results suggest that key length selection materially impacts end-to-end latency through the cryptographic stages—especially cloud-side decryption—while leaving the iterative solver’s stability intact. Consequently, practical deployments should calibrate key lengths to balance privacy budget/security requirements against latency and prioritize parallel/throughput optimizations for CSP decryption to mitigate the dominant bottleneck under higher key sizes.

Per-round computation and communication are as follows (1024-bit modulus unless noted). Each user produces three encryptions per round (distance; $w_{t}^{u}x_{t}^{u}$; $w_{t}^{u}$), at ≈16–17 ms/encryption, i.e., ≈50 ms per user per round (Table 3 and Table 4, “Client HE”). Per task, the FN’s ciphertext products cost ≈3–4 ms/round (“FN multiply”), while the CSP performs three decryptions per round (for ∑d, ∑wx, ∑w) at ≈14–15 ms/decryption, i.e., ≈45 ms per task per round (“CSP decrypt”). CSP decryption is the dominant bottleneck, scaling near-linearly with the number of tasks and rounds. Communication per Paillier ciphertext is 2∣n∣ bits; for a 1024-bit modulus, this is 2048 bits ≈ 256 B. Thus, user→FN traffic is ≈768 B per user, per round (three ciphertexts), and FN→CSP traffic is ≈768 B per task, per round (three aggregated ciphertexts). Key length sensitivity (Table 5) shows steep growth due to big-integer arithmetic—encrypt/decrypt times of ≈2.37/1.73 ms (512-bit), ≈16.80/14.50 ms (1024-bit), and ≈108.56/95.27 ms (2048-bit)—while convergence remains stable (~9–10 rounds), indicating that larger keys mainly affect latency rather than solver stability.

To assess the effect of repeated runs on efficiency stability, this experiment repeats the task-wise private pipeline 10 times under a fixed workload (20 dimensions, 135 users) to quantify run-to-run stability of end-to-end latency and stage costs. Table 6 summarizes the per-run measurements (total runtime in minutes; stage times in milliseconds). The total runtime averages 30.50 min with a standard deviation of 1.52 min (CV ≈ 4.98%), indicating stable end-to-end latency across runs. Per-stage distributions show small dispersion as well: Client HE averages 18.03 ms (CV ≈ 5.45%), FNs multiply 3.93 ms (CV ≈ 6.23%), and CSPs decrypt 14.40 ms (CV ≈ 6.43%). The update phases are also consistent—weight update averages 2451.44 ms (CV ≈ 5.96%) and truth update 4826.56 ms (CV ≈ 4.98%). The box plots highlight tight inter-run variability for each stage, and the stacked bar of stage means confirms the bottleneck composition observed in earlier sections: CSP decryption and client-side encryption dominate the cryptographic overhead, while fog node multiplication is comparatively minor. Taken together, these results demonstrate that under a fixed sensing workload, the proposed pipeline exhibits low run-to-run variance in both total and per-stage costs, reinforcing its robustness and repeatability under identical settings.

**Figure 8:**
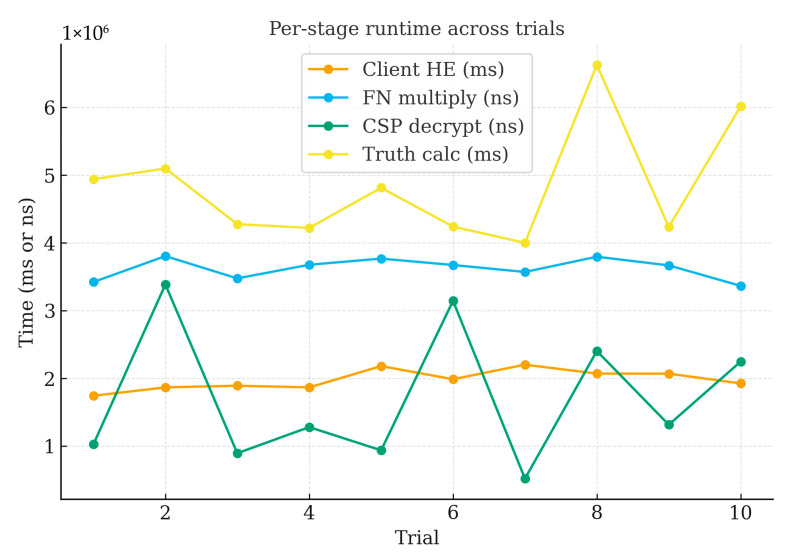
Per-stage runtime across trials (key length 1024).

Figure 8 summarizes per-stage runtime across 10 independent trials under identical settings (1024-bit modulus). The client-side homomorphic encryption and the CSP decryption stages dominate latency, whereas the FN ciphertext multiplication remains comparatively small and stable. Trial-to-trial variability is limited and exhibits no trend, which aligns with the narrow confidence intervals. These observations are consistent with the stage breakdowns in Table 3, Table 4 and Table 5 and support the identification of CSP decryption as the principal bottleneck under increasing task/user cardinalities and larger key sizes.

**Figure 9:**
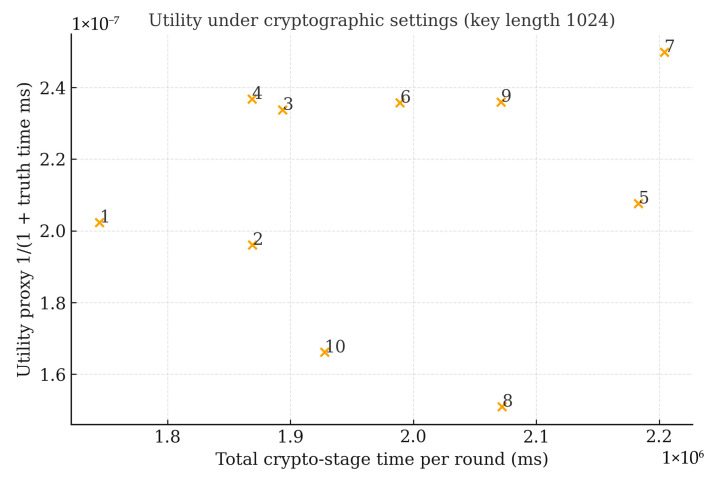
Utility under cryptographic settings.

Moreover, Figure 9 plots total cryptographic-stage time per round (client HE + FN multiply + CSP decrypt, in milliseconds) against a utility proxy derived from the truth-calculation time (1/(1+truth_time)) under the 1024-bit setting. The scatter shows that utility remains essentially flat over small latency fluctuations, consistent with the observation that homomorphic aggregation affects latency but not aggregation fidelity. When results for multiple key lengths are available (e.g., 512/1024/2048 bits), the same figure can be rendered as a key length curve (latency rising steeply; MAE/RMSE remaining nearly constant), highlighting the latency–security trade while preserving accuracy.

## 5. Conclusions

This work presents a fine-grained, personalized aggregation scheme for mobile crowd sensing that integrates truth discovery with structural privacy protections. User–task weights and task truths are updated in an alternating schedule until a normalized convergence criterion is satisfied, while aggregation is carried out under Paillier with aggregate-only decryption and task-scoped unlinkable pseudonyms. Under this design, both location information and raw measurements remain protected, and task-wise weighting preserves aggregation fidelity relative to a non-private oracle while enabling personalized incentive settlement.

The proposed scheme attains structural privacy through Paillier aggregate-only decryption and task-scoped pseudonyms, yet several constraints remain that readers should bear in mind. Trust is still concentrated in centralized roles, leaving residual exposure to single-point failure and potential insider misbehavior despite hardware protection. The adversary model is largely semi-honest, so coordinated or targeted poisoning could exploit the reweighting loop with format-correct but false reports and thereby bias the discovered truths. Privacy is argued by construction and formal definitions rather than by empirical leakage measurement, and implementation-level side channels have not been assessed. Operational choices—pseudonym lifecycle, replay protection, tamper-evident logging, and parameters—introduce overhead and sensitivity that have not yet been benchmarked across device and network conditions.

Looking ahead, distributing trust via t-of-m threshold Paillier or MPC can remove any single entity’s unilateral decryption capability, and multi-authority pseudonym issuance can further reduce linkage risk; these directions complement the current minimal-disclosure design without altering its aggregation logic. To strengthen input integrity and robustness, zero-knowledge range/consistency proofs can constrain submitted distances and weights without revealing raw data, while robust aggregators and lightweight anomaly screens over encrypted or minimally revealed statistics can improve resistance to poisoning and collusion. A ledger-backed audit and incentive layer is also promising for decentralized accountability and verifiable, fine-grained rewards. Future evaluations will include leakage audits and systematic latency/energy/throughput studies under stronger collusion and adversary models to guide practical deployment of personalized, privacy-preserving truth discovery.

## Figures and Tables

**Table 1:** Privacy/security properties under collusion patterns.

Collusion Pattern	C1 Raw-Claim Confidentiality	C2 Weight–Identity Unlinkability	C3 Correctness	C4 Auditability
None (baseline)	✓	✓	✓	✓
CSP↔FN	✓	✗	✓	✓
CSP↔TA	✓	✓	✓	✓
FN↔Users	✗	✓	✓	✓
TA↔CSP↔FN	✗	✗	✓	✓

“✓” = The property remains secure under this type of collusion. “✗” = The property is weakened or invalidated under this type of collusion.

**Table 2:** The accuracy and convergence of two weighting strategies.

Method	MAE	RMSE	Max Iters
Task-wise private	1.33 × 10^−5^	1.39 × 10^−5^	12
Global/unified private	0.0417	0.0529	37

**Table 3:** Runtime and convergence across dimensions.

Dimensions	Total Runtime (min)	Client HE (ms)	FN Multiply (ms)	CSP Decrypt (ms)	Iterations
5	7.8957	16.7584	4.0685	12.1474	9
10	13.3567	15.5703	3.6292	13.1995	9
15	20.7321	16.6370	3.8232	13.1432	10
20	28.1068	16.8028	3.7169	14.4956	9

**Table 4:** Runtime and convergence across user counts.

Users	Total Runtime (min)	Client HE (ms)	FN Multiply (ms)	CSP Decrypt (ms)	Iterations
25	6.98	15.977	0.705	13.536	14
45	9.64	15.284	1.162	11.919	9
90	17.71	15.129	2.331	12.118	10
135	28.1068	16.8028	3.7169	14.4956	9

**Table 5:** Runtime and convergence across key lengths.

Key Length (bits)	Total Runtime (min)	Client HE (ms)	FN Multiply (ms)	CSP Decrypt (ms)	Iterations
512	3.94	2.369	1.397	1.728	9
1024	28.11	16.803	3.717	14.496	9
2048	179.59	108.559	10.654	95.272	10

**Table 6:** Per-run measurements 10 times.

Metric	Mean	Std	Min	Max	CV%
Total runtime (min)	30.50	1.52	27.92	32.47	4.98
Client HE (ms)	18.030	0.98	16.75	19.12	5.45
FN multiply (ms)	3.93	0.24	3.49	4.22	6.23
CSP decrypt (ms)	14.40	0.93	13.26	15.75	6.43
Weight update (s)	2.45	0.15	0.23	0.27	5.96
Truth update (s)	4.83	0.24	0.46	0.52	4.98

## Data Availability

This article uses public datasets.

## Abbreviations

The following abbreviations are used in this manuscript:

MCS	Mobile Crowd Sensing
TA	Trust Authority
CSP	Cloud Service Platform
FNs	Fog Nodes
